# Glucose-insulin-potassium alleviates uterine cramping pain following cesarean delivery: A randomized, controlled trial

**DOI:** 10.3389/fsurg.2022.1068993

**Published:** 2023-01-09

**Authors:** Guiying Yang, Yu Cui, Xiaohang Bao, Zhuoxi Wu, Qin Chen, Feng Chen, Wenjun Liu, Mingming Wang, Li Luo, Hong Li

**Affiliations:** ^1^ Department of Anesthesiology, Second Affiliated Hospital of Army Medical University, Chongqing, China; ^2^ Department of Anesthesiology, the Affiliated Hospital, School of Medicine, UESTC Chengdu Women’s & Children’s Central Hospital, Chengdu, China; ^3^ Department of Gynaecology and Obstetrics, Second Affiliated Hospital of Army Medical University, Chongqing, China

**Keywords:** analgesia, cesarean delivery, glucose-insulin-potassium, postpartum, uterine cramping pain single-center, randomized controlled study

## Abstract

**Objectives:**

To explore the effect of glucose-insulin-potassium (GIK) therapy on uterine cramping pain (UCP) following cesarean delivery (CD).

**Design:**

Single-center, randomized controlled study.

**Setting:**

Second Affiliated Hospital of Army Medical University, Chongqing, China.

**Participants:**

A total of 140 women, aged 20–40 years, who underwent CD with a transverse incision were randomly assigned to the GIK (P) or control (C) groups in a 1:1 ratio.

**Interventions:**

GIK was intravenously administered to patients in Group P. Patients in Group C received normal saline (NS). After umbilical cord clamping, oxytocin was administered intravenously. The same GIK and NS regimens were administered on postoperative days 1 and 2, followed by oxytocin 10 min later.

**Primary and secondary outcome measures:**

Following oxytocin administration, UCP was assessed using the visual analog scale (VAS), and the maximum VAS score (primary outcome) was recorded.

**Results:**

Patients in Group P had significantly lower maximum VAS scores than those in Group C on postoperative days 1 (38.4 ± 21.1 vs. 52.3 ± 20.8, *p* < 0.001) and 2 (10 [0,30] vs. 30.5 [8.75,50], *p* < 0.001). Group P patients also had shorter pain duration on postoperative day 1 (39.6 ± 19.5 min vs. 50.6 ± 18.2 min, *p* = 0.001). Group P patients had a lower incidence of inadequate analgesia of UCP than Group C on days 1 (45.5% vs. 74.2%, *p* < 0.001) and 2 (10.6% vs. 47.0%, *p* < 0.001); the RRs for experiencing inadequate analgesia for UCP postpartum in Group P patients was 0.612 (95% CI: 0.454–0.826, *p* < 0.001) on day 1 and 0.226 (95% CI: 0.107–0.476, *p* < 0.001) on day 2. The absolute risk reduction (ARR) was 28.7%; thus number needed to treat (NNT) was 3 after rounding up. A subgroup analysis demonstrated that Group P patients undergoing repeat CD had lower maximum VAS scores for UCP on both postoperative days 1 and 2.

**Conclusion:**

Our findings suggest that GIK can relieve UCP and shorten its duration. Our results provide information to facilitate the development of novel approaches for managing UCP.

**Clinical Trial Registration:** This study was approved by the Medical Ethics Committee of Second Affiliated Hospital of Army Medical University (2020-109-01, 19/11/2020) and registered in the Chinese Clinical Trial Registry (http://www.chictr.org.cn, ChiCTR2100041607,01/01/2021).

## Introduction

Cesarean delivery (CD) is the most common inpatient surgery worldwide (1) but remains associated with severe postoperative pain (2). A major cause of analgesic infectivity postoperatively is uterine cramping pain (UCP) (3). UCP is exacerbated by oxytocin, which is routinely used to reduce uterine bleeding following CD.

In China, it is routine to use oxytocin for more than 2 days following CD which aggravates UCP in puerperas (4, 5). The Society for Obstetric Anesthesia and Perinatology recommends a multimodal pain management plan for CD patients consisting of long-acting neuraxial opioid analgesia and scheduled nonsteroidal anti-inflammatory drugs (NSAIDs) or acetaminophen. Peripheral regional nerve block is reserved only for unique cases (6). Patient-controlled epidural analgesia provides effective but suboptimal outcomes due to the side effects which include motor block (7) and urinary retention (8). Patient-controlled opioid intravenous analgesia (PCIA) following CD allows pain control without these complications. Intravenous opioid analgesics, including sufentanil which is used in PCIA, however, have a poor effect on UCP (9) and intrinsic side effects associated with opioids (10). It is, therefore, imperative to identify novel therapies for UCP which can provide optimal pain relief with limited side effects.

Uterine ischemia/hypoxia is one of the mechanisms underlying UCP (11–13). A study in mice demonstrated that the uterine visceral pain pathways include altered muscle contractility and impaired perfusion (14). An oxytocin-induced increase in uterine pressure reduced uterine oxygenation by 38% in the absence of inflammatory molecules. Further, the use of platelet-activating factors which induce uterine contractions can reduce uterine perfusion by 40 ± 8%, elicit oxygen desaturation levels close to hypoxia (9.4 ± 3.4 mm Hg PaO_2_), and produce visceral pain. By improving tolerance to uterine ischemia/hypoxia and metabolism, UCP may be relieved and, therefore, physiological treatment strategies for UCP warrant studying.

Physiological treatments have been successful in the treatment of cardiac-related pain or angina. Angina is caused by cardiac ischemia/hypoxia resulting in the accumulation of lactate, 5-hydroxytryptamine, bradykinin, and prostaglandin (15). Similarly, uterine contraction causes ischemia/hypoxia and lactate accumulation (16), which are crucial mechanisms of pain. Angina and UCP may have similar mechanisms of pain and respond to similar treatments. In this regard, Dmitrovic et al. (17) reported that dysmenorrhea may be alleviated by increasing uterine blood flow using the vasodilator nitric oxide.

One treatment for angina is glucose-insulin-potassium (GIK). This is used clinically to increase myocardial metabolism (18), protect against acute myocardial ischemia, improve cardiac function (19), and relieve or delay angina (20, 21): It does so by promoting glucose uptake and utilization (22), increasing glycolysis, and effectively clearing lactate (23, 24). We hypothesized that GIK may improve uterine energy metabolism, reducing the accumulation of pain-causing molecules and helping relieve UCP symptoms. This randomized clinical trial aimed to explore the effect of GIK therapy on postpartum UCP following CD.

## Materials and methods

### Patients

This single-center, randomized controlled study was approved by the Institutional Ethics Committee of the Second Affiliated Hospital of Army Medical University (ID:2020-109-01). The study has been registered in the Chinese Clinical Trial Registry (http://www.chictr.org.cn, ChiCTR2100041607). All patients provided written informed consent before enrollment.

**Figure 1:**
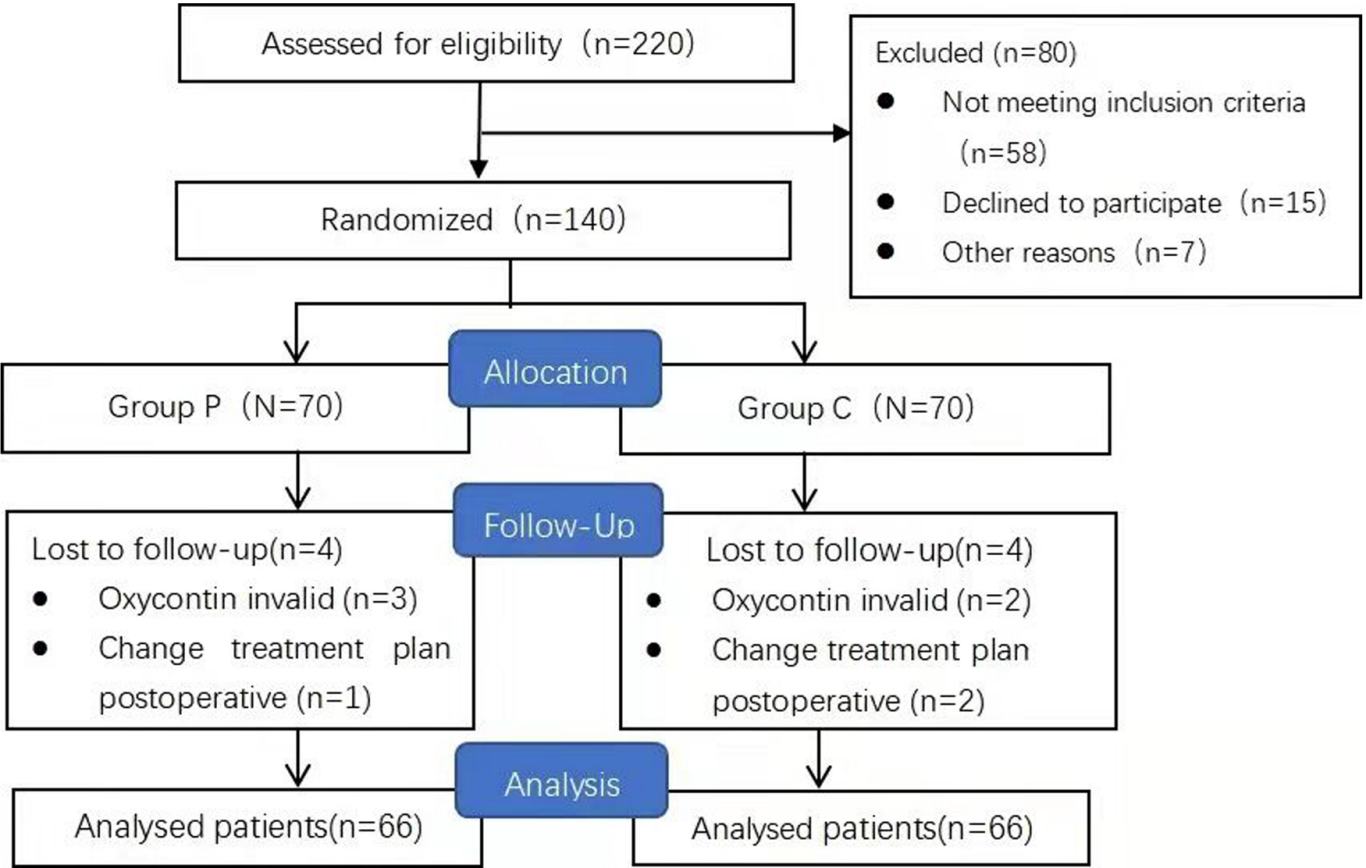
Study flow diagram.

Between January and March 2021, we enrolled 140 women aged 20–40 years who were scheduled to undergo CD with a transverse incision (Figure 1). The inclusion criteria were as follows: a full-term gestation of 37–40 weeks, single pregnancy, an American Society of Anesthesiologists physical status scale of I–II, acceptance of the administration of subarachnoid anesthesia, and voluntary reception of intravenous PCIA. The exclusion criteria included: moderate or worse anxiety [Generalized Anxiety Disorder Questionnaire (GAD-7) score >9], depression [Patient Health Questionnaire-9 (PHQ-9) score >9], gestational diabetes mellitus, severe hypertension or eclampsia, placenta previa, chronic pain, long-term use of analgesics, and renal insufficiency. Withdrawal criteria included participants who did not experience uterine contraction and UCP (VAS score consistently 0 at all assessments) following oxytocin administration, postoperative changes in the treatment plan, and lactation during oxytocin effect after oxytocin administration.

### Anesthesia and analgesia management

All patients agreed to receive subarachnoid anesthesia [Ropivacaine (AstraZeneca AB, 20–25 mg)] by an experienced anesthesiologist. This was initiated immediately following CD. PCIA was administered using an electronic infusion pump containing 800 mg tramadol (Sandoz Pharmaceutical Co. Ltd., China) in 0.9% normal saline (NS, 300 ml). The background volume was 6.0 ml/h, and the patient-controlled analgesia (PCA) dose was 2 ml, with a locking time of 15 min. Acetaminophen and NSAIDs were not administered, and tramadol was the only rescue analgesic for inadequate analgesia.

### Drug intervention program

Participants were randomly assigned to the GIK (Group P) or control (Group C) groups. For Group P patients, GIK (250 ml of 10% glucose, 8 IU of insulin, and 0.8 g of KCl) was intravenously (IV) administered at a rate of 1.5 ml/kg/h when patients entered the operating room. For patients who did not complete GIK infusion intraoperatively, infusion was continued postoperatively. Group C received NS at the same dose and rate. Per the hospital's protocol, oxytocin (20 U in 100 ml of 0.9% normal saline) was administered as an IV continuous infusion immediately after umbilical cord clamping. Blood potassium and glucose levels were measured *via* arterial blood gas analysis before and after GIK or NS infusion on the day of surgery. On the morning of days 1 and 2 following CD, GIK or NS was infused using the described regimen, and IM oxytocin (10 U) was injected 10 min after infusion. The GIK composition and injection rate were based on prior studies (25, 26). The onset of insulin IV is approximately 15–30 min post-injection, whereas the onset of oxytocin IM is approximately 5 min. IM oxytocin was administered 10 min following GIK infusion with the expectation that the two drugs would take effect simultaneously.

### Randomization and masking

We performed stratified randomization based on primary or repeat CD (the main factors affecting UCP) using the sealed envelope method and a ratio of 1:1. A biostatistician generated random numbers using IBM SPSS Statistics for Windows, version 26.0 (IBM Corp., Armonk, NY). The pain anesthesiologist prepared the distribution sequence and placed the numbers in sealed envelopes. Participants were randomly allocated into two groups with different intervention strategies (GIK or NS infusion) in the operating room before CD. The envelopes were re-sealed and kept blinded until the end of the study. GIK or NS were administered using the same infusion bag to ensure that patients and researchers could not distinguish between the medications. Although pain associated with injection might have led to unblinding for researchers, we attempted to ensure blinding to the best of our ability in this study. The pain management anesthesiologist was not involved in data collection or analysis. Preoperative and intraoperative data were collected by one anesthesiologist. A nurse anesthetist collected postoperative follow-up data during the hospitalization period and 1-week postoperatively through telephone interviews.

### Outcome measurements

All data were collected from the medical records of the Second Affiliated Hospital of Army Medical University. Demographic characteristics including height, weight, age, preoperative complications such as anemia and subclinical thyroid dysfunction, dysmenorrhea, gestational age, and sleep quality in the week before the operation were collected. All participants completed the GAD-7 [each item scored on a 0–3 scale (total score, 0–21)] and PHQ-9 [each item scored on a 0–3 scale (total score, 0–27)] questionnaires to assess their anxiety and depression status, respectively. Information regarding operation time, fluid infusion volume, concomitant surgeries such as tubal ligation, uterine fibroids or ovarian cyst surgery, intrauterine balloon for uterine inertia, and intraoperative bleeding volume were recorded.

### Primary outcomes

The primary outcome was the maximum UCP visual analog scale (VAS) scores measured during oxytocin use on the morning of postoperative days 1 and 2. VAS scores (0–100, where 0 and 100 were defined as no and maximum pain, respectively) were used to assess UCP following IM oxytocin injection at 10-min intervals. The nurse anesthetist remained with the patients at all times, verbally used the VAS scale to assess their pain level, assessed the maximum UCP every 2 min, and recorded their maximum VAS score as the maximum UCP over 10 min.

### Secondary outcomes

The secondary outcomes included UCP and incisional pain before oxytocin administration, time of onset time, and duration of oxytocin use. We also assessed the period of maximum incision pain, UCP VAS following oxytocin initiation, and number of PCA applications during oxytocin treatment on postoperative days 1 and 2. Postoperative blood loss (12 h) and height of the uterine fundus (distance from the uterine fundus to the lower edge of the umbilicus assessed by a nurse anesthetist) were also recorded (27). We recorded the time of lactation commencement, i.e., the time until the first drop of breast milk was expressed. UCP was also assessed on postoperative day 7 using the numerical rating scale (NRS, 0–10, where 0 was defined as no pain and 10 as maximum pain) through a telephone interview.

The specific definitions for pain measurement were as follows:

• The onset time was defined as the time between IM oxytocin injection and the onset of UCP. If a patient already had symptoms of UCP, the onset time was defined as the onset of increased intensity or frequency of UCP.
• The duration time was defined as the time between IM oxytocin injection and the resolution of UCP. If a patient had UCP before oxytocin administration, the duration end-point was defined as the point at which the UCP intensity and frequency returned to pre-administration levels.
• The periods of maximum incisional pain and UCP on postoperative day 1 were the maximum VAS scores for incisional pain and UCP from the end of surgery to oxytocin injection on postoperative day 1.
• The periods of maximum incision pain and UCP on postoperative day 2 were the maximum VAS scores for incision pain and UCP from the resolution of UCP on postoperative day 1 to the time of oxytocin injection on postoperative day 2.
• The maximum VAS score for UCP referred to the maximum VAS score from the first oxytocin injection to the resolution of UCP after postoperative day 2.
• Inadequate analgesia for UCP was defined as a VAS score ≥40 during oxytocin treatment.
• The number of PCA applications referred to the number of PCA button presses between the first oxytocin injection and the resolution of UCP after postoperative day 2.

### Sample size calculation

Based on our preliminary study (*n* = 20) of patients receiving routine clinical treatment, the main outcome measured was the maximum UCP VAS score during oxytocin use on postoperative day 1. The mean maximum UCP VAS score was 35.9 ± 22.9. The maximum UCP VAS score was expected to decrease with GIK therapy, and the minimum clinical between-group difference was 0.5 standard deviations (11.45). With the significance level and power set at 0.05 and 0.9, respectively, the minimum sample size required was 128. The sample size was calculated using PASS version 11.0 (NCSS, LLC. UT, USA). Approximating a loss of follow-up rate of 10%, we eventually included 140 patients.

### Statistical analysis

Statistical analyses were performed using IBM SPSS Statistics for Windows, version 26.0. Statistical significance was set at *p* < 0.05. Continuous variables are presented as the mean (SD), number (frequency), and median (interquartile range).

A stepwise logistic regression analysis was used to evaluate the role of demographic, preoperative, intraoperative data to predict factors associated with inadequate analgesia of UCP. All variables in Table 1, primiparas or multiparas status, and the use of GIK were included in this model. Odds ratio (OR) with a 95% confidence interval (CI) was calculated.

**Table 1:** Demographic, preoperative, and intraoperative data.

	Group P *n* = 66	Group C *n* = 66	*p* value
Age (years)	30.1 ± 4.0	31.4 ± 3.1	0.646
Height (cm)	159.0 ± 5.4	158.5 ± 5.1	0.585
Weight (kg)	69.6 ± 10.1	69.3 ± 9.2	0.871
Sleep quality NRS score in 1 week preoperative	5 (3,7)	5 (5,6)	0.414
PHQ-9 (score)	1 (0,1)	1 (1,1)	0.052
GAD-7 (score)	0 (0,1)	0 (0,2)	0.349
Gestation age (week)	38.8 ± 1.0	38.2 ± 2.2	0.440
Dysmenorrhea	26 (39.5%)	22 (33.3%)	0.469
Intrauterine balloon tamponade	11 (16.7%)	10 (15.2%)	0.812
Infusion volume (ml)	798.0 ± 280.6	795.0 ± 292.0	0.962
Blood loss volume (ml)	335.6 ± 134.4	328.0 ± 134.5	0.747
Surgery duration (min)	93.5 ± 27.3	88.0 ± 19.3	0.121
Synchronized other surgery	7 (10.6%)	7 (10.6%)	1.000

Data are presented as means (SD), numbers (frequency), or median (interquartile range). PHQ, patient health questionnaire [each item scored on a 0–3 scale (total score: 0–27)]; GAD, anxiety disorder questionnaire [each item scored on a 0–3 scale (total score: 0–21)]; NRS, numerical rating scale (NRS score, 0–10, where 0 and 10 denote good and poor sleep quality, respectively).

An independent single-sample t-test was used for the between-group comparisons of normally distributed demographic and perioperative data. The Mann–Whitney *U* test was used for between-group comparisons of non-normally distributed data, including PHQ-9 score, GAD-7 score, onset time, height of the uterine fundus, number of PCA applications, VAS score for incisional pain and UCP before oxytocin, maximum VAS score for UCP during oxytocin administration, duration time on postoperative day 2, and UCP NRS on postoperative day 7. Pearson's chi-square test or Fisher's exact test were used for between-group comparisons of categorical data, including preoperative complications, dysmenorrhea, other concomitant surgeries, intrauterine balloon tamponade, inadequate analgesia for UCP during oxytocin treatment, and incisional pain. We calculated the relative risk (RRs) and 95% CI for inadequate analgesia of UCP during oxytocin treatment, and absolute risk reduction (ARR) and number needed to treat (NNT) were also calculated. A two-way repeated analysis of variance (ANOVA) was used to analyze between-group differences in blood glucose and blood potassium levels.

## Results

We initially recruited 140 patients of which eight were excluded for deviation from the study protocol (Figure 1). At the end of the study, 132 patients were included in the analyses. Since a stratified randomization strategy depending on primary or repeat CD was utilized, 33 primary and 33 repeat CD patients were included in both Groups C and P. No significant between-group differences were found in the preoperative and intraoperative parameters (*p* > 0.05) (Table 1).

### Risk factor analysis

As summarized in Table 2, for all included participants, the results of stepwise logistic regression analysis showed that multiparas status was a risk factor for inadequate analgesia of UCP on postoperative 1 day (OR: 2.075, 95% CI: 1.012–4.215, *p* = 0.046). Sleep quality NRS score 1 week preoperative was also a risk factor for inadequate analgesia of UCP on postoperative 2 day (OR: 1.345, 95% CI: 1.038–1.755 *p* = 0.025). The use of GIK was identified as a protective factor for inadequate analgesia of UCP on postoperative days 1 and 2 (OR: 0.298, 95% CI: 0.141–0.630, *p* = 0.002 and OR: 0.100, 95% CI: 0.034–0.298, *p* < 0.001, respectively).

**Table 2:** Factors associated with inadequate analgesia in a stepwise logistic regression analysis.

Outcome	Factors with statistical significance	Wald	*p* value	OR	95% CI
Inadequate analgesia of UCP on postoperative day 1	Primiparas or multiparas	3.975	0.046	2.075	1.012–4.251
	The use of GIK	10.066	0.002	0.298	0.141–0.630
Inadequate analgesia of UCP on postoperative day 2	Sleep quality NRS score in 1 week preoperative	5.017	0.025	1.350	1.038–1.755
	The use of GIK	18.088	<0.001	0.100	0.034–0.298

CI, confidence interval; OR, odds rate; UCP, uterine cramping pain; NRS, numerical rating scale (NRS score, 0–10, where 0 and 10 denote good and poor sleep quality, respectively).

### VAS scores on postoperative days 1 and 2

**Figure 2:**
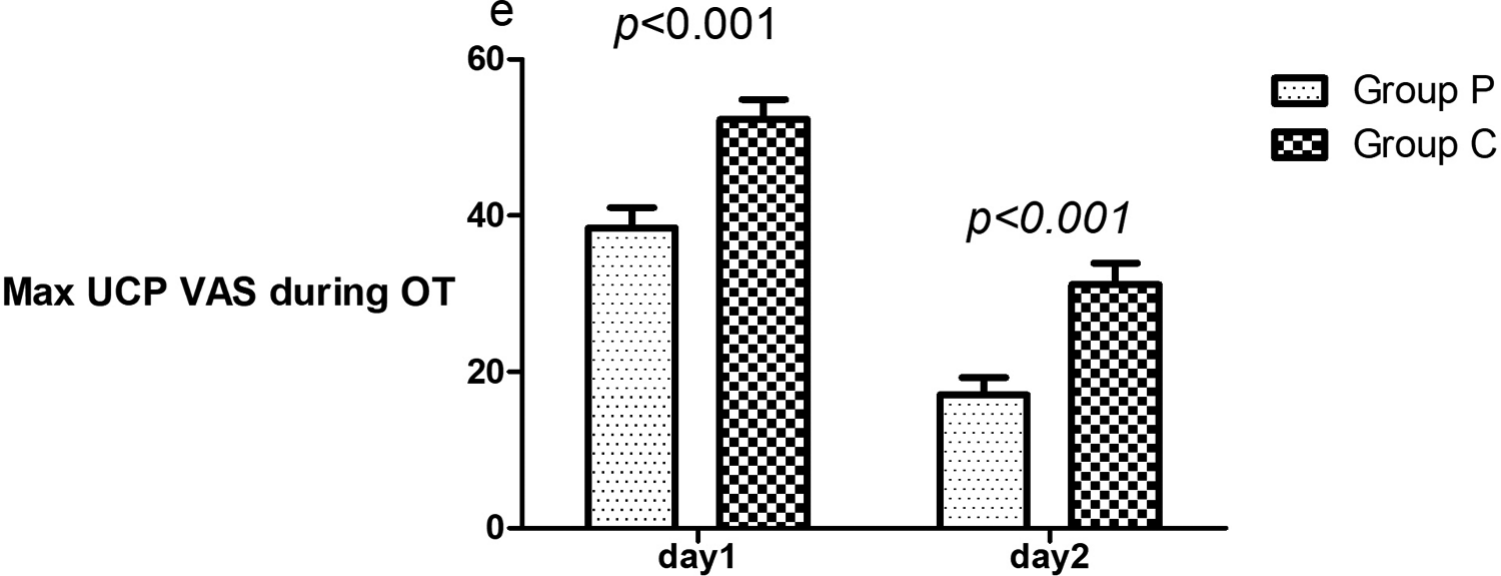
Comparison of maximum UCP VAS during oxytocin on days 1 and 2. The mean scores of patients in Groups P and C who underwent GIK and NS. VAS, visual analog scale (0–100, where 0 and 100 denote no and maximum pain, respectively); UCP, uterine cramping pain; OT, oxytocin; Max, maximum; GIK, glucose–insulin–potassium; VAS, visual analog scale.

Compared to patients in Group C, Group P patients had a lower maximum VAS score for UCP during oxytocin use on days 1 (38.4 ± 21.1 vs. 52.3 ± 20.8, *p* < 0.001) and 2 (10 [0,30] vs. 30.5 [8.75,50], *p* < 0.001) (Figure 2).

### Secondary outcomes on postoperative days 1 and 2

Group P patients had a lower incidence of inadequate analgesia of UCP than Group C on days 1 (45.5% vs. 74.2%, *p* < 0.001) and 2 (10.6% vs. 47.0%, *p* < 0.001); the RRs for experiencing inadequate analgesia for UCP postpartum in Group P patients was 0.612 (95% CI: 0.454–0.826, *p* < 0.001) on day 1 and 0.226 (95% CI: 0.107–0.476, *p* < 0.001) on day 2. The ARR was 28.7% (incidence of inadequate analgesia was 45.5% in group P and 74.2% in group C); thus NNT was 3 after rounding up. Additionally, Group P patients had a shorter duration of UCP than group C patients (39.6 ± 19.5 min vs. 50.6 ± 18.2 min, *p* = 0.001) on day 1 (Table 3).

**Table 3:** Secondary outcomes on postoperative days 1 and 2.

	Postoperative day 1			Postoperative day 2		
	Group P *n* = 66	Group C *n* = 66	*p* value	Group P *n* = 66	Group C *n* = 66	*p* value
Onset time of oxytocin (min)	7 (5,9)	5 (4,8)	0.184	7 (5,10)	7 (5,10)	0.815
Inadequate analgesia of UCP	30 (45.5%)	49 (74.2%)	<0.001	7 (10.6%)	31 (47.0%)	<0.001
Duration time of oxytocin (min)	39.6 ± 19.5	50.6 ± 18.2	0.001	25 (5,38)	30 (15,48)	0.143
Incision pain VAS before oxytocin	0 (0,22)	0 (0,10)	0.227	0 (0,0)	0 (0,0)	0.964
UCP VAS before oxytocin	40.5 ± 16.6	44.1 ± 18.0	0.237	29.4 ± 14.0	34.8 ± 17.6	0.054
Time period of max incision pain VAS postoperative	78.5 ± 18.6	78.1 ± 18.2	0.906	48.1 ± 20.1	56.1 ± 19.3	0.084
Time period of max UCP VAS postoperative	43.9 ± 26.6	48.4 ± 27.0	0.237	0 (0,20)	0 (0,25.75)	0.977
Number of PCA applications	0 (0,0)	0 (0,0)	0.368	0 (0,0)	0 (0,0)	1.000
Height of the uterine fundus (cm)	1 (0,2)	1 (0,2)	0.802	1 (0,2)	1 (0,2)	0.944
Incidence of injection pain of GIK	36 (54.5%)	0 (0%)	<0.001			
Injection pain VAS of GIK	0 (0,10)	0 (0,0)	<0.001			
Blood loss at postoperative 12 h (ml)	66.5 ± 20.6	57.8 ± 19.1	0.014			
Start lactation time postoperative (h)	13 (1,20)	16 (5,36)	0.047			

Data are presented as means (SD), numbers (frequency), or median (interquartile range). UCP, uterine cramping pain; PCA, patient-controlled analgesia; VAS, visual analogs scale (0–100, where 0 and 100 indicate no and maximum pain, respectively); GIK, glucose-insulin-potassium.

Group P patients had a higher incidence of injection pain than Group C patients (54.5% vs. 0.0%, *p* < 0.001) (Table 2). Patients in Group P had less blood loss 12 h after CD than patients in Group C (57.7 ± 19.1 ml vs. 66.4 ± 20.6 ml, *p* = 0.014). Group P patients also had a shorter time to lactation than Group C (patients 13[1,20] h vs.16[5,36] h, *p* = 0.047). No significant between-group differences were found in the uterine fundus height (*p* > 0.05) and UCP NRS on postoperative day 7 (0[0,0] vs. 0[0,0], *p* = 0.664).

### Subgroup analysis for primary and repeat CD patients

Subgroup analysis revealed that Group P patients had a lower maximum UCP VAS score on postoperative days 1 and 2 than Group C patients in repeat CD patients (39.8 ± 18.8 vs. 59.2 ± 17.9; 10 [0,29] vs. 23 [0,36], respectively, *p* < 0.001). No significant difference was noted in the maximum UCP VAS score in primary CD patients (Table 4).

**Table 4:** Subgroup analysis for primary and repeat cesarean delivery on maximum UCP on postoperative days 1 and 2.

Number of cesarean delivery		Group P	Group C	*p* value
Primary cesarean delivery	Maximum UCP VAS postoperative 1 day	37.27 ± 23.0	45.4 ± 21.4	0.135
	Maximum UCP VAS postoperative 2 day	16 (0,30)	21 (5,50)	0.064
Repeat cesarean delivery	Maximum UCP VAS postoperative 1 day	39.8 ± 18.8	59.2 ± 17.9	<0.001
	Maximum UCP VAS postoperative 2 day	10 (0,29)	23 (0,36)	<0.001

Data are presented as means (SD) or as median (interquartile range). UCP, uterine cramping pain; VAS, visual analog scale (0–100, where 0 and 100 indicate no and maximum pain, respectively).

### Effect of blood glucose and potassium levels on Maximum UCP

Blood glucose and potassium levels were measured before and after administration of GIK or NS on postoperative days 1 and 2. A two-way repeated ANOVA revealed no significant time, group, or interaction effects on potassium levels (*p* > 0.05). Blood glucose levels showed a significant time effect (*p* < 0.001); however, no significant group or time and group interaction effects were noted (*p* > 0.05). Blood glucose levels in Group P patients increased after medication (5.6 ± 1.0 vs.4.5 ± 0.6, *p* = 0.018), but no significant difference was observed in Group C patients.

## Discussion

Our results show that GIK reduced patients’ UCP VAS score and pain duration during oxytocin treatment following CD. We also found GIK-related pain improvement to be significantly better in patients undergoing repeat CD and GIK to promote early lactation. Compared to controls, group P patients displayed a higher incidence of injection pain. Our findings suggest that GIK treatment improves pain in patients with CD but remains associated with slight injection pain.

Anai et al. (28) reported that lactate dehydrogenase deficiency in pregnant women decreased adenosine triphosphate production during anaerobic glycolysis in the myometrium and increased UCP. Glycolysis is an important energy supply in the uterine contraction process. Its metabolic disorders cause uterine inertia and UCP, suggesting that glycolysis can improve uterine energy metabolism and relieve UCP. A study on rats in the late trimester of pregnancy reported an increase in myometrial glycogen levels, an enhancement in glycogen decomposition for high-energy supply during labor, greater myometrial sensitivity to insulin, and increased glucose uptake in the presence of insulin (29). We observed that GIK decreased patients’ UCP VAS score and duration after CD, suggesting that glucose and insulin are critically involved in supplying energy for uterine contraction. As mentioned, GIK can stimulate myocardial glycolysis and clear lactate to relieve angina. The mechanisms underlying UCP and angina relief by GIK may be similar. We suspect that glucose in GIK may provide energy for uterine contraction and insulin can increase myometrial glucose and substrate metabolism. Additionally, GIK may stimulate glycolysis, increase high-energy phosphate levels, and effectively clear pain metabolites.

We found the pain treatment effect of GIK to be most apparent in patients undergoing repeat CD. A previous study has shown that multiparas experience more severe UCP (30), and other studies have suggested that UCP intensity is related to ischemia of the uterus and myometrial blood flow (11, 12). We speculate that GIK is more effective for patients with severe uterine contractions and severe ischemia. Nevertheless, the effects of GIK on glycolysis, local metabolites of the myometrium, and the relationship between the GIK analgesic effect and uterine contraction intensity need further investigation.

Pain inhibits the secretion of prolactin and oxytocin, delaying the start of lactation and reducing output (31). Glucose is an important energy substrate that is required for the initiation of lactation (32). Patients treated with GIK had increased blood glucose levels and an earlier lactation onset time than patients given saline. GIK-induced acceleration of lactation may contribute to UCP relief and increased blood glucose levels.

This study has several limitations. First, to minimize trauma to patients without diabetes, changes in blood sugar and potassium levels were monitored only before and after GIK or NS infusion on the day of surgery. Second, we did not monitor uterine local blood flow and metabolism, since there is no non-invasive method to accurately do so. Currently available methods are unable to penetrate the abdominal wall tissue because of the excessive thickness of maternal abdominal fat. Third, the optimal proportion, dosage, and rate for GIK infusion could not be established in this study and further studies are necessary. Fourth, this study may have lacked total blinding due to injection pain experienced by 54% of GIK recipients compared to 0% of control recipients. Finally, this study may lack generalizability to some postpartum populations as the experimental conditions implemented in this study were artificial due to IM oxytocin on postpartum days 1 and 2 to generate UCP, and these patients received no intrathecal opioids, acetaminophen, or NSAIDs for pain control.

## Conclusion

In conclusion, our findings suggest that GIK can alleviate UCP and shorten its duration. Treatments that improve uterine tolerance to ischemia/hypoxia may effectively treat UCP. Our findings may provide a novel approach to UCP treatment. Multimodal pain management, however, remains the mainstay for CD analgesia. Scheduled acetaminophen and non-steroidal drugs were the basic drugs, and long-acting neuraxial opioid analgesia or other intravenous analgesia should be also used. When UCP following CD is not effectively controlled using standard multimodal analgesia regimens, GIK may be used as an auxiliary method for UCP control.

## Data Availability

The raw data supporting the conclusions of this article will be made available by the authors, without undue reservation.
